# Gender stereotypes in agreement processing with role nouns: a study on Russian

**DOI:** 10.3389/fpsyg.2025.1619505

**Published:** 2025-09-17

**Authors:** Natalia Slioussar, Daria Antropova

**Affiliations:** ^1^School of Linguistics, Higher School of Economics, Moscow, Russia; ^2^Centre for Language and Brain, Higher School of Economics, Saint Petersburg, Russia

**Keywords:** grammatical gender, gender stereotypes, agreement processing, nouns denoting professions and social roles, Russian

## Abstract

The majority of Russian nouns denoting professions and social roles are grammatically masculine. Some of them have feminine pairs, the others do not, but in modern Russian, most nouns in this group can be used to refer to women — either with masculine or with feminine agreement. This option has some interesting limitations that have been extensively discussed in different theoretical approaches (feminine agreement is grammatical only in the nominative; some combinations of feminine and masculine agreement are ungrammatical). However, very few studies are dedicated to processing and acceptability of the sentences with such nouns. To fill this gap, we conducted three experiments: two word-by-word self-paced reading studies and one acceptability judgment study. Following previous studies of role nouns in different languages, we focused on the interaction of grammatical and extralinguistic factors: grammatical gender in attributive and predicative agreement and gender stereotypes associated with different professions and social roles. We revealed a clear preference for masculine agreement both offline (despite the fact that feminine agreement is grammatical) and online, although it was less pronounced for the sentences with stereotypically “female” professions. In general, ungrammatical sentences had the lowest ratings and the longest reading times, although in the sentences with stereotypically “male” professions, feminine agreement was so unexpected that it could slow down reading times more than ungrammaticality. In some other respects, offline and online data showed curious differences: sentences in which the gender of the predicate matches the gender of the attributive adjective were read significantly faster, but did not receive higher ratings.

## 1 Introduction

Russian nouns have three grammatical genders: masculine, feminine and neuter (M, F, and N). Like many other languages, Russian faces the following problem: historically, many nouns denoting professions and social roles were grammatically masculine. In the modern world, these professions and roles also became available to women. How to call a female director or a female author?

In Russian, two routes are available. Firstly, a corresponding grammatically feminine noun can be formed (e.g., *žurnalist* ‘journalist_M_’ – *žurnalistka* ‘journalist_F_’, *učitel’* ‘teacher_M_’ – *učitel’nica* ‘teacher_F_’).^1^ Unlike German, where the *-in* suffix can be attached to most relevant nouns, and like French, Russian uses a variety of suffixes to form such nouns. However, many masculine role nouns do not have an established feminine pairs, and for many others, feminine derivates are considered colloquial.

Secondly, Russian has so-called common gender nouns that can be used with masculine and feminine agreement, like *plaksa* “crybaby_F/M_”. In modern Russian, this option became available to any role noun, although with many interesting limitations that provoke heated debates in the theoretical literature — we will discuss them in the next section. Cross-linguistically, this is an unusual pattern. As we show in the next section, a number of studies explores the acquisition of gender in this group of nouns, which is significantly delayed compared to most other nouns. However, very few analyze how they are processed with masculine and feminine agreement. In the present paper, we aim to fill this gap. As we know from studies on various languages, the processing of role nouns is influenced by gender stereotypes associated with them. Therefore, we decided to focus on the interaction between stereotypicality and morphosyntactic factors. This will let us shed new light on a more general question: how grammatical and extralinguistic factors interact at different processing stages.

The paper has the following structure. In the next section, we present the complex system of genders and inflectional classes (declensions) of Russian nouns, highlighting the controversies that surround role nouns. After that, we overview processing studies focusing on these nouns in different languages, which show a central role of gender stereotypes associate with them. In last section of the introduction, we set the goals for the present study, and then three experiments we conducted are presented.

### 1.1 Gender and declension of Russian nouns with a focus on role nouns

Russian nouns are inflected for six cases and two numbers. They may have different sets of affixes depending on their inflectional class, or declension. Traditional reference grammars (e.g., Shvedova, 1980), as well as many other studies (e.g., Aronoff, 1994; Halle, 1994), identify three declensions with several subparadigms and various exceptions. We will rely on the model with three declensions outlined in Table 1 for the sake of convenience because our findings do not allow teasing different approaches apart. Let us also note that in plural, most differences between declensions disappear. Moreover, Russian has gender agreement only in singular: on adjectives, participles and past tense verb forms. Therefore, in this paper, we will focus on singular forms.

**Table 1:** A system of four inflectional classes for Russian nouns.

Classes	Descriptions	Examples	Percentage of nouns in the RNC
Declension I	F nouns ending in *-(j)a* in nominative singular	*komnata* ‘room’, *zemlja* ‘earth’	29%
	M nouns (only denoting people) ending in -*(j)a* in nominative singular	*papa* ‘dad’, *djadja* ‘uncle’	1%
Declension IIa	M nouns ending in a consonant or in a soft sign in nominative singular	*zakon* ‘law’, *kon’* ‘horse’	46%
Declension IIb	N nouns ending in *-o* or -*e* in nominative singular	*okno* ‘window’, *more* ‘sea’	18%
Declension III	F nouns ending in a soft sign in nominative singular	*kost’* ‘bone’	5%
Indeclinable	nouns of different genders with different endings	*kivi* ‘kiwi’, *pal’ to* ‘coat’	1%

Percentages of nouns in the Russian National Corpus, or RNC (www.ruscorpora.ru), are taken from Slioussar and Samojlova (2015). Their counts were based on the grammatically disambiguated subcorpus and did not take substantivized adjectives into account. There is also a very small number of exceptional cases with irregular inflection. In Russian, *ja* and *ju* are separate letters, although they are transliterated as combinations of two symbols. The symbol*’* stands for the letter *soft sign* that usually indicates that the preceding consonant is palatalized, but has a number of other functions. i.e., phonologically, all nouns in the classes IIa and III end in a consonant and have a zero inflection in nominative singular (in other cases, they have overt inflections: e.g., *zakona, zakonu* etc. from *zakon* “law”).

Table 1 shows that the gender of the noun cannot be unambiguously determined from its inflectional affix, but there is a strong correlation between the two. The absolute majority of nouns ending in *-(j)a* (declension I) are feminine, although there is a small group of masculine nouns (some of them highly frequent) and an even smaller group of common gender nouns that was not included in Table 1. Both “exceptional” groups contain only nouns denoting people. Common gender nouns usually denote personal qualities (e.g., *plaksa* “crybaby_F/M_” or *umnica* “smart person_F/M_”), but sometimes also professions and social roles (e.g., *sudja* ‘judge_F/M_’). Nouns that end in a soft sign in nominative singular may be masculine (declension IIa) or feminine (declension III). In most cases, their gender can be determined only from agreeing adjectives, participles and verb forms or from their own forms in oblique cases: e.g., *gelja, gelem* etc. from *gel’* ‘gel_M_’ or *meli, mel’ju* etc. from *mel’* ‘sandbank_F_’.

Historically, all declinable nouns that end in a consonant in nominative singular are masculine (declension IIa). Most role nouns belong to this group (e.g., *psixolog* “psychologist_M/(F)_”) and constitute an interesting exception. Firstly, in modern Russian they can be used to refer to a woman, unlike other animate masculine nouns (denoting personal qualities, ethnicities and nationalities etc.). Remarkably, this is possible even when a feminine noun exists, like in the pair *učitel’* ‘teacher_M_’ – *učitel’nica* “teacher_F_”.^2^ Secondly, when referring to a woman, they may be used either with feminine or with masculine agreement in the nominative (termed *semantic* and *formal agreement*), but only with masculine agreement in oblique cases (e.g., Muchnik, 1971; Graudina et al., 1976; Zaliznjak, 2002).

More recent studies noted that some examples of feminine agreement in oblique cases are found in real usage (e.g., Savchuk, 2011; Sitchinava and Chuprinko, 2022), while Magomedova and Slioussar (2021) conducted a more detailed corpus study and several experiments analyzing how such cases are processed. Although some interesting differences between oblique forms were found, Magomedova and Slioussar do not dispute the claim that only nominative forms are fully acceptable with feminine agreement. The authors argue that this is due to the strong correlation between genders and declensions: Russian has other feminine nouns with a zero inflection in the nominative (declension III), but all oblique case inflections do not coincide in these declensions. A similar claim was later made by Privizentseva (2024). In their word-by-word self-paced reading study, Magomedova and Slioussar (2021) included both oblique and nominative case forms and found that not only in the former, but also in the latter case feminine agreement is processed more slowly, although for nominative, this delay is very local.

Except for Magomedova and Slioussar (2021), all existing experimental studies on Russian focus on nominative forms, with which both masculine and feminine agreement is grammatical. We will also do so in the present study. Several early experiments (Panov, 1968; Novikov and Priestly, 1999) analyzed the choice of gender in verbs and attributive adjectives agreeing with nouns denoting professions and social roles. They concluded that semantic agreement is more frequent with verbs than with adjectives. Moreover, examples like (1), in which the adjective shows feminine agreement and the verb is in masculine, are ungrammatical, while all other combinations are possible (ungrammatical sentences are marked with asterisk). Further studies revealed other interesting patterns, like certain distinctions between qualitative and relative adjectives. These phenomena were discussed in different theoretical frameworks (e.g., Asarina, 2009; Caha, 2019; Corbett, 1982, 1991, 2006, 2023; Lyutikova, 2015; Matushansky, 2013; Pesetsky, 2013; Privizentseva, 2024; Steriopolo, 2019; Steriopolo and Wiltschko, 2010). All authors agree that syntactically, verbs (and other predicates) are further away from the agreement controller than attributive adjectives, and the further away the agreement target is, the higher the chances for semantic agreement, although the implementations of this idea differ depending on the framework. We will not further discuss different theoretical approaches to this phenomenon, because our experimental data do not let us tease them apart.

(1) *Sedaja pedagog družeski poxlopal vypusknika po pleču.

grey-haired_F_ teacher friendly tapped_M_ graduate on shoulder
‘The grey-haired teacher friendly tapped the graduate on the shoulder.’

Russian role nouns also attracted attention in the field of language acquisition: several authors demonstrated that semantic agreement is acquired relatively late (e.g., Dizer, 2007; Dobrova, 2013; Rodina and Westergaard, 2012; Rodina, 2014; Tseitlin, 2000). However, very few studies explore how sentences with these nouns are processed. We aim to fill this gap paying special attention to gender stereotypes associated with these nouns — as we show in the next section, they were found to affect processing in different languages, interacting with grammatical factors.

### 1.2 Processing sentences with role nouns: the influence of gender stereotypes

Many processing studies dedicated to role nouns focused on the influence of an important extralinguistic factor: gender stereotypes associated with different roles and professions. Gender stereotypes are viewed as a part of real-world knowledge. The general question how real-world knowledge affects sentence processing bears upon the debate between constructivist (e.g., Graesser et al., 1994) and minimalist (e.g., McKoon and Ratcliff, 1992) approaches. The former suggest a crucial role of inferences in comprehension, while the latter claim that they are limited. Garnham (2005) and Garnham and Yakovlev (2015) claimed that gender stereotypes associated with role nouns are a vivid example of inferences made during processing, since information about stereotypicality was shown to be invoked immediately (e.g., Banaji and Hardin, 1996; Garnham et al., 2002; Oakhill et al., 2005; Reynolds et al., 2006; Pyykkönen et al., 2010).

Information about gender stereotypes in these experiments was taken from surveys conducted by the authors themselves or independently (e.g., Gabriel et al., 2008). In such surveys, participants are usually asked to answer how many men and women a particular professional group contains. These surveys were conducted separately for speakers of different languages, although the results are largely parallel. Other characteristics of their participants (their age, educational level etc.) have not been shown to produce significant effects so far.

A number of studies focused on the acceptability of sentences explicitly referring to men or women given the context with role nouns in the plural form, like in (2). In the languages in which nouns have grammatical gender (like in French, Spanish, German or Norwegian, unlike in English), masculine nouns can be used as *generic plurals:* to refer to a group of people including both men and women, or to people whose gender is unknown or considered unimportant. In English, gender stereotypes associated with role nouns were found to influence acceptability judgments: reference to women was judged as more acceptable in the context of the female stereotype, while reference to men was more acceptable in the context of the male stereotype (Gygax et al., 2008; Garnham et al., 2012). Gygax et al. (2008) did not observe a similar effect for French and German. They only noted that masculine plural forms, despite being used as generic plurals, make a subsequent reference to women less acceptable. However, a later study by Garnham et al. (2012) found a stereotypicality effect in both languages: the male bias of generic plurals was more pronounced for stereotypically “male” role nouns. The same pattern was observed in Spanish (Anaya-Ramírez et al., 2022). Stereotypicality effects were also reported for Norwegian (Gabriel and Gygax, 2008; Gabriel et al., 2017), in which both male and female stereotypes influenced acceptability ratings.

(2) *The social workers were walking through the station. Since sunny weather was forecast several of the women were not wearing a coat*. (Gygax et al., 2008)

Another group of studies was dedicated to the influence of stereotypicality on online reading measures. In English, mismatches between the pronoun *he/she* and the stereotype of the antecedent, like in (3), caused delays in reading times (Carreiras et al., 1996; Kennison and Trofe, 2003). The same was observed for reflexive pronouns *himself/herself* (Sturt, 2003; Duffy and Keir, 2004). In Spanish, sentences with mismatches between the gender of a role noun and gender stereotypes associated with it, as in (4a), were read significantly slower than congruent sentences like (4b) (Carreiras et al., 1996). However, after this mismatch was processed it did not influence reading times in the following sentences. Thus, the pronoun *él* ‘he’ was read after (4a) as fast as the pronoun *ella* ‘she’ after (4b).

(3) *The electrician examined the light fitting. She needed a special attachment to fix it.* (Carreiras et al., 1996)
(4) a. *El enfermero tuvo que suturar la herida*. (Carreiras et al., 1996)

‘The_M_ nurse_M_ had to suture the injury.’
b. *La enfermera tuvo que suturar la herida*. (Carreiras et al., 1996)
‘The_F_ nurse_F_ had to suture the injury.’

Irmen (2007) conducted an eye-tracking study on German including paired role nouns (e.g., *Schreiner* “carpenter_M_” – *Schreinerin* “carpenter_F_”) and common gender role nouns (e.g., *Haushaltsangestellte* “domestic employee_M/F_”). When masculine paired nouns were used generic plurals, a subsequent reference to women (*diese Frauen* “these women”) was costlier than a reference to men (*diese Männer* “these men”). The effect became more pronounced in the context of a male stereotype. This was in line with the male bias and its interaction with stereotypicality observed in acceptability judgments (Garnham et al., 2012). For common gender role nouns, a mismatch between stereotypes associated with them and a subsequent noun phrase used anaphorically induced a processing cost. The effect of male stereotypes was visible in early and late measures, while the effect of female stereotypes was observed only in late measures. In another eye-tracking study on German (Esaulova et al., 2014), mismatches between gender stereotypes and pronouns or noun phrases used anaphorically increased the frequency of regressions to the latter. The effect was more pronounced for noun phrases and for male stereotypes.

The only processing study focusing on stereotypicality effects with role nouns in Russian was conducted by Garnham and Yakovlev (2015). They also collected a set of stereotype norms for Russian role nouns. Since their study is especially relevant for our project, we will discuss it in some detail. Firstly, relying on previous work on other languages (Gabriel et al., 2008; Kennison and Trofe, 2003; Misersky et al., 2014), Garnham and Yakovlev composed a list of 160 Russian masculine role nouns. Using various dictionaries, the nouns were divided into three groups: (1) 44 nouns that have an established feminine counterpart (e.g., *student* ‘student_M_’ – *studentka* ‘student_F_’); (2) 55 nouns without a feminine counterpart (e.g., *èlektrik* ‘electrician_M_’)’; (3) 61 nouns with a colloquial feminine counterpart (e.g., *vrač* ‘doctor_M_’ – *vračixa* ‘doctor_F_’). Previous studies on other languages, like Irmen (2007), demonstrated that pairedness may be important for processing of role nouns.

All role nouns selected by Garnham and Yakovlev end in a consonant and can be used to refer to women. The authors mention that Russian has a small number of other role nouns: some common gender nouns like *sudja* “judge_F/M_”, a couple of feminine nouns that do not have a masculine pair like *njanja* “nanny_F_”, several pairs like *medbrat* “nurse_M_” – *medsestra* “nurse_F_”, in which the masculine noun cannot be used to refer to a woman (which is probably due to the fact that these words are derived from the nouns *brat* “brother_M_”, *sestra* “sister_F_” and an abbreviated adjective “medical”). However, these “atypical” role nouns were not included in the study. Let us also mention that pairedness is a complicated issue in Russian. Some paired feminine nouns that exist only in colloquial Russian are more frequently used, the others are less, some are clearly derogative, like *vračixa* ‘doctor_F_’, while the others, quite on the contrary, are mostly used by the speakers who support feminist values, like *avtorka* “author_F_”. Since in our study, we used only nouns that do not have established feminine pairs, we will not discuss this problem in more detail here (we selected nouns from Garnham and Yakovlev’s list, which is based on several dictionaries, and also relied on our own native speaker intuitions).

After 160 role nouns were selected, Garnham and Yakovlev (2015) conducted a rating study. 106 speakers of Russian recruited via social networks took part in it. 87 were female, 16 were male, and three participants chose not to specify their gender, i.e., the distribution was unbalanced. Garnham and Yakovlev do not provide information about their age and educational level, which is unfortunate, although we do not know of any studies showing how such characteristics may affect the results. In every trial, the participants were presented with a noun in the nominative plural form and asked to imagine a large group of people denoted by this noun (e.g., students, electricians or doctors). The task was to indicate which proportion of men and women is likely to be present in this group, using an 11-point scale (0% women and 100% men, 10% women and 90% men, and so on, up to 100% women and 0% men). Based on average ratings, nouns were grouped into denoting stereotypically ‘male’, stereotypically ‘female’ or neutral professions. In general, stereotypicality norms were similar to those observed in other languages.

In the experimental part of the study, Garnham and Yakovlev (2015) used passages consisting of two sentences, as in (5a-b). In the first sentence, role nouns were used as subjects (the authors selected nouns from the stereotypically male, stereotypically female and neutral groups, with established feminine pairs or without them). The second sentence started with a feminine or masculine personal pronoun. Verbs could be in the present or in the past tense in both sentences. In the past tense, verbs show gender agreement, and the two verb forms were always matched in this respect both with each other and with the gender of the pronoun. Sentence-by-sentence reading times were measured, and after reading every passage, the participants were asked to provide a ‘yes/no’ judgment whether the sentences constitute a sensible text.^3^

(5) a. Present tense:

*Kosmetolog govorit po telefonu. Ona/On ob’jasnjaet novomu klientu, kak ix najti.*
‘The beautician is talking on the phone. She/he is explaining to a new client how to find them.’
b. Past tense:
*Kosmetolog govorila/govoril po telefonu. Ona/On ob’jasnjala/ob’jasnjal novomu klientu, kak ix najti.*
‘The beautician talked_F/M_ on the phone. She/He explained_F/M_ to a new client how to find them.’

Garnham and Yakovlev (2015) observed that first sentences with feminine agreement were read slower, and this factor interacted with stereotypicality. In the second sentence, stereotypicality interacted with the gender of the pronoun. These results were in line with stereotype mismatch effects reported for other languages. No significant effects for acceptability judgments were reported.

Interestingly, in the previous studies (e.g., Carreiras et al., 1996), a mismatch between stereotypes and the referent’s gender caused immediate delays and did not affect later processing. In the study by Garnham and Yakovlev (2015), the effect of the mismatch, which became apparent on the verb in the past tense, persisted to the second sentence (although did not always reach significance). The researchers suggested that the gender of the verb may play a weaker role in the representation of the referent’s gender than the gender of a definite article (in Spanish). We would refine this explanation: in Russian, both masculine and feminine agreement with role nouns is grammatical when referring to women, which indeed makes the gender of the predicate a weaker cue.

### 1.3 The present study

The overview in the previous section shows how processing of sentences with role nouns is affected by gender stereotypes associated with these nouns, which can be observed both in acceptability judgments and in online reading measures. Experiments on languages with grammatical gender suggested that stereotypicality can interact with grammatical information. However, previous studies focused on the gender of pronouns and noun phrases used anaphorically, while Russian also allows analyzing gender agreement with role nouns. Garnham and Yakovlev (2015) included it in their experiment, but measured only sentence-by-sentence reading times and acceptability of two-sentence passages (which contained both verb forms and pronouns).

In the present paper, we use more fine-grained online and offline measures to study how different factors influence agreement processing with role nouns.^4^ We conducted three experiments: two of them measured word-by-word reading times, while the third one measured acceptability of target sentences on the 1 to 5 scale. As a result, we could analyze the interaction of extralinguistic and grammatical factors at different stages of agreement processing.

In Experiment 1, we compared how masculine and feminine agreement on the predicate is processed with role nouns (stereotypically “female” vs. stereotypically “male”) and with nouns denoting personal qualities. As we noted in section 1.1, most nouns in the latter group have a pair of the opposite gender, e.g., *krasavec* “beautiful person_M_” – *krasavica* “beautiful person_F_”, masculine nouns cannot be used to refer to a woman, masculine agreement is grammatical with one noun in the pair, and feminine with the other. We hypothesized that, although feminine agreement is grammatical with role nouns, it may be more difficult to process than masculine agreement (potentially, also depending on gender stereotypes). Therefore, we wanted to compare these cases to some sentences in which only masculine or only feminine agreement is grammatical.

In Experiment 2, we added attributive adjectives to the picture. The readers first saw a masculine or feminine adjective, then a target role noun, and a masculine or feminine verb form, followed by some words depending on the predicate. We wanted to find out whether participants would rely not only on stereotypes, but also on the grammatical gender of the adjective when processing the gender of the verb. In Experiment 3, we collected acceptability judgments for the sentences used in Experiment 2 to compare online processing and offline ratings.

## 2 Experiment 1

The goal of this experiment was to find out how grammatical features and extralinguistic knowledge (stereotypes associated with different professions and social roles) influence subject–verb agreement processing with role nouns in Russian.

### 2.1 Participants

62 native speakers of Russian (28 male and 34 female) aged 18–50 took part in Experiment 1. No participant took part in more than one experiment. In all experiments, participants were university students or people with higher education with no linguistic background. We recruited them online via social networks, they received no compensation for their participation and volunteered out of curiosity. All experiments reported in this paper were carried out in accordance with the Declaration of Helsinki and the existing Russian and international regulations concerning ethics in research. All participants were naïve to the experimental hypotheses and provided informed consent.

### 2.2 Materials

We created 32 sets of target sentences, as in (6a–b), (7a–b), and (8a–d). All sentences were six words long and had the same syntactic structure: a subject noun – a copula (*byt’* ‘to be’ in the past tense) – an adjective or participle – three words modifying the predicate. All subjects and predicates were in the singular, predicates had feminine or masculine agreement. We opted for predicates with copulas because they were used in many previous studies of agreement processing in Russian (e.g., Slioussar and Malko, 2016; Slioussar, 2018; Slioussar et al., 2022). Target sentences were divided in two groups. In Group 1, subjects were role nouns. Using the list created by Garnham and Yakovlev (2015), we selected eight stereotypically female professions, as in (6a–b), and eight stereotypically male professions, as in (7a–b).^5^ All these nouns do not have established feminine pairs — as we showed in section 1.2, this factor may affect processing. In Group 2, subjects were paired nouns denoting personal qualities. Thus, examples (8b) and (8d) are ungrammatical.

(6) Group 1F: stereotypically female (SF) professions

a. SFM: *Pediatr byl obespokoen iz-za objavlenija karantina.*
pediatrician was_M_ worried_M_ because (of) announcement (of) quarantine
b. SFF: *Pediatr byla obespokoena iz-za objavlenija karantina.*
pediatrician was_F_ worried_F_ because (of) announcement (of) quarantine
  ‘Pediatrician was worried because of the announcement of the quarantine.’

(7) Group 1 M: stereotypically male (SM) professions

a. SMM: *Mjasnik byl soglasen s povarom stolovoj.*
butcher was_M_ accrodant_M_ with cook (of) canteen
b. SMF: *Mjasnik byla soglasna s povarom stolovoj.*
butcher was_F_ accrodant_F_ with cook (of) canteen
‘The butcher agreed with the cook of the canteen.’

(7) Group 2: personal qualities.

a. MM: *Intrigan byl ostorožen v ètom voprose.*
intriguer_M_ was_M_ cautious_M_ in this question
b. *MF: *Intrigan byla ostorožna v ètom voprose.
intriguer_M_ was_F_ cautious_F_ in this question
c. FF: *Intriganka byla ostorožna v ètom voprose.*
intriguer_F_ was_F_ cautious_F_ in this question
d. *MF: *Intriganka byl ostorožen v ètom voprose.
intriguer_F_ was_M_ cautious_M_ in this question
‘The (male or female) intriguer was cautious in this question.’

Thus, the following parameters were manipulated: (i) the gender of the predicate; (ii) whether the subject noun refers to a stereotypically female or male profession (in Group 1F vs. Group 1 M); (iii) whether the subject is feminine or masculine (in Group 2). Subject nouns in Groups 1F and 1 M are grammatical with feminine agreement, but morphologically resemble masculine nouns, so feminine agreement may be less expected after them, especially in Group 1 M.^6^ Our goal was to determine whether this is reflected in processing. We compared Groups 1F and 1 M between each other and to the examples in Group 2 to find out how similar sentences with feminine agreement like (6b) and (7b) are to grammatical sentences like (8c) or to ungrammatical sentences like (8b), and in what ways they differ from the corresponding sentences with masculine agreement.

We distributed target sentences across four experimental lists using the Latin square principle (the sentences from the groups 1F and 1 M were the same in two lists). The task also featured 64 grammatically correct filler sentences that did not contain role nouns or common gender nouns. The full list of stimuli can be found at OSF.^7^ For one third of the target and filler sentences, we created forced choice comprehension questions to ensure that the participants were reading properly. An example is given in (9).

(9) *Cto bespokoit pediatra?*

‘What worries the pediatrician?’

1. karantin
‘quarantine’
2. èpidemija
‘epidemics’

### 2.3 Procedure

In all experiments reported in this paper, before proceeding to the main part, participants read the instructions and some information about the study, filled in a small questionnaire about their gender, age and education, and provided informed consent to participate in the study. After that, three practice items were presented. Each practice sentence was followed by a question.

The sentences were presented on a PC using the Ibex Farm platform.^8^ We used word-by-word self-paced reading methodology. Each trial began with a sentence in which all words were masked with dashes while spaces remained intact. Participants were pressing the space bar to reveal a word and re-mask the previous one. Word-by-word reading times were recorded.

The order of target and filler sentences was fully randomized. One third of the sentences was followed by comprehension questions. The question and two answer variants were presented one above the other, as in (9). Participants pressed ‘1’ to choose the answer above, and ‘2’ to choose the answer below. Participants were instructed to read at a natural pace and to answer questions as accurately as possible. They were not informed in advance that some sentences would contain errors.

### 2.4 Analysis

We analyzed participants’ question-answering accuracy and reading times. On average, participants answered only 5.3% of questions incorrectly (13.6% at most). Given the low number of mistakes, a breakdown of RTs into correct and incorrect question trials was not performed. Reading times that exceeded a threshold of 2.5 standard deviations, by region and condition, were excluded (Ratcliff, 1993). In total, 3.4% of the data was excluded (at most 6.0% per region and condition).

We modeled the data with mixed-effects linear regressions in the *R* software^9^ using the *lmer* function from the *lme4* package (Bates et al., 2015). To obtain *p* values from the *t* values given by the model, we used the *lmerTest* package (Kuznetsova et al., 2017). Random intercepts by participant and by item were included in the model (we tried building models with random intercepts and random slopes, but they did not converge).

The group (1F, 1 M, or 2), predicate gender (masculine or feminine) and grammaticality (in Group 2: grammatical or ungrammatical) were treated as fixed effects in different comparisons. We also included two-way interactions between the factors. For the predicate gender and grammaticality, we used contrast coding. Masculine subject was coded as 0, feminine as 1; grammatical was coded as 0, ungrammatical as 1. When all three groups were compared, Group 2 was used as the reference level. When Group 1F was compared to 1 M, 1F was coded as 0 and 1 M as 1.

### 2.5 Results and discussion

**Figure 1:**
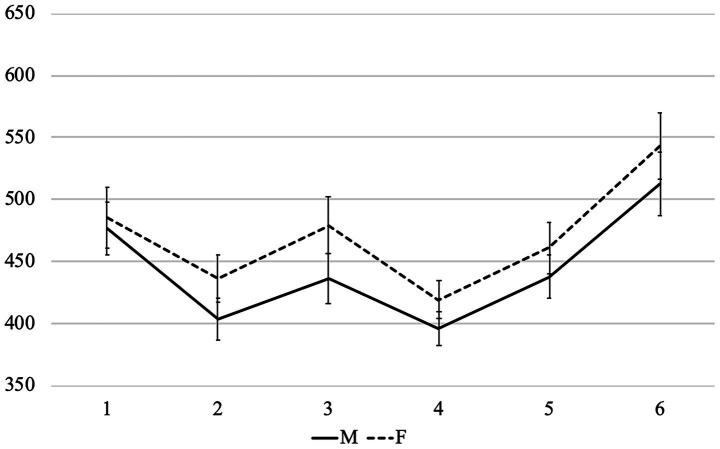
Average word-by-word RTs (in ms) in the group 1M (with nouns denoting stereotypically male professions).

**Figure 2:**
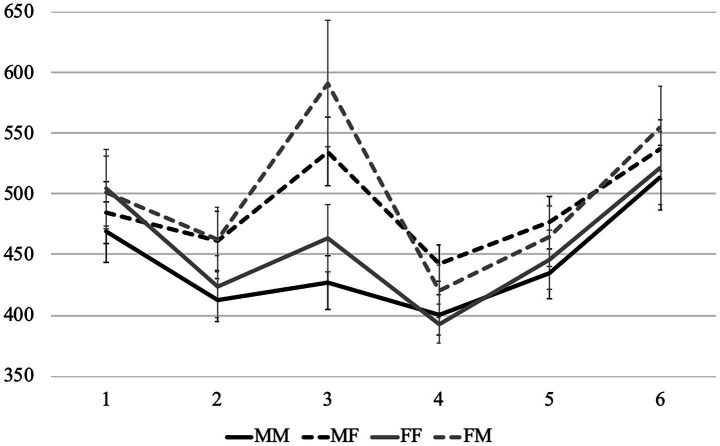
Experiment 1: Average word-by-word RTs (in ms) in the group 2 (with nouns denoting personal qualities).

**Figure 3:**
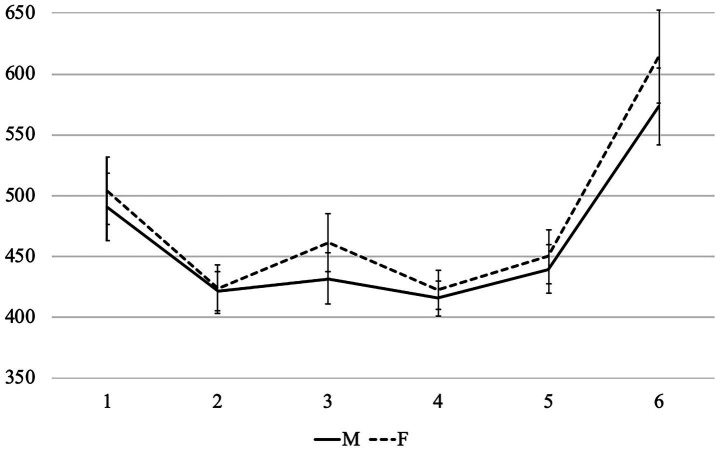
Experiment 1: Average word-by-word RTs (in ms) in the group 1F (with nouns denoting stereotypically female professions).

Average reading times per region in different conditions are presented in Figures 1–3. Before turning to the statistical analysis, let us note that sentences with feminine agreement take longer to read than those with masculine agreement in Group 1F and especially in Group 1 M. But these differences are less pronounced than the differences between grammatical and ungrammatical sentences in Group 2.

In all comparisons, there were no significant effects in region 1 (the subject noun), as expected, and we did not analyze the last word of the sentence (region 6) because the results would be difficult to interpret due to wrap-up effects. Therefore, we focus on regions 2–5 below. Only statistically significant results are reported, while full outputs of all models can be found at OSF.

Firstly, we analyzed sentences in Group 2 with predicate gender and grammaticality as fixed effects. Group 2 shows how masculine and feminine agreement are processed when either one or the other can be expected after the subject noun. Only the grammaticality factor was significant (region 2: *β =* 50.91, *SE =* 14.64, *t =* 3.48, *p <* 0.001; region 3: *β =* 106.15, *SE =* 19.85, *t =* 5.35, *p <* 0.001; region 4: *β =* 42.97, *SE =* 10.11, *t =* 4.25, *p <* 0.001; region 5: *β =* 44.98, *SE =* 12.26, *t =* 3.67, *p <* 0.001). Thus, we cannot say that feminine agreement is intrinsically more difficult to process with any (animate) noun — this would be important for the analysis of role nouns.

Then we analyzed sentences in Groups 1F and 1 M with predicate gender and group, i.e., stereotypicality, as fixed effects (these two groups differ in terms of gender stereotypes associated with subject nouns). In region 2, the interaction between the factors was significant (*β =* 34.55, *SE =* 13.56, *t =* 2.55, *p =* 0.01), i.e., the delay associated with feminine agreement was more pronounced in Group 1 M than in Group 1F. In region 3, the predicate gender factor was significant (*β =* 27.11, *SE =* 11.32, *t =* 2.40, *p =* 0.02): feminine agreement was processed more slowly. We can conclude that, although both masculine and feminine agreement was grammatical in these two groups, sentences with feminine agreement took longer to process — probably due to the fact that subject nouns morphologically resemble masculine nouns. Gender stereotypes also affected processing — very early, starting from the first word showing gender agreement. Feminine agreement was easier to process after subject nouns denoting stereotypically female roles and professions.

After that, we compared grammatical sentences in all three groups with predicate gender and group as fixed effects. In region 3, the predicate gender factor was significant (*β =* 34.34, *SE =* 12.03, *t =* 2.85, *p <* 0.01): sentences with feminine agreement had longer reading times. In region 4, the interaction between predicate gender and Group 1 M reached significance (*β =* 28.22, *SE =* 11.95, *t =* 2.36, *p =* 0.02). This shows that the delay associated with feminine agreement in Group 1 M was significantly larger than in Group 2, which can be viewed as the baseline, but there was no such difference between Group 1F and Group 2. In other words, only sentences with stereotypically male roles and professions significantly differed from other grammatical sentences with feminine agreement.

Since subject nouns in Groups 1F and 1 M resemble masculine nouns, we also compared sentences from these groups to sentences with masculine subjects from Group 2, using predicate gender and group as fixed effects. We aimed to find out how feminine agreement in Groups 1F and 1 M differs from ungrammatical feminine agreement in Group 2. The predicate gender factor was significant in regions 2–5 (region 2: *β =* 51.34, *SE =* 10.75, *t =* 4.78, *p <* 0.001; region 3: *β =* 106.97, *SE =* 12.20, *t =* 8.77, *p <* 0.001; region 4: *β =* 42.26, *SE =* 8.23, *t =* 5.13, *p <* 0.001; region 5: *β =* 42.22, *SE =* 10.42, *t =* 4.05, *p <* 0.001): feminine agreement took longer to process. The interaction between predicate gender and Group 1F reached significance in the same four regions (region 2: *β =* −51.35, *SE =* 15.21, *t =* −3.38, *p <* 0.001; region 3: *β =* −80.02, *SE =* 17.19, *t =* − 4.66, *p <* 0.001; region 4: *β =* −34.52, *SE =* 11.64, *t =* −2.97, *p <* 0.01; region 5: *β =* −35.65, *SE =* 14.68, *t =* −2.43, *p =* 0.02). In other words, feminine agreement with nouns denoting stereotypically female roles and professions always triggered a significantly smaller delay than ungrammatical feminine agreement. The interaction between predicate gender and group 1 M also reached significance, but only in region 3 (*β =* −63.60, *SE =* 17.24, *t =* −3.69, *p <* 0.01). Thus, even in case of male stereotypes, feminine agreement with role nouns causes smaller delays than ungrammatical feminine agreement, although the effect is less pronounced than in case of female stereotypes.

To summarize the findings, gender stereotypes play an important role in online agreement processing. Sentences with feminine agreement in Group 1 M differed significantly both from grammatical and from ungrammatical examples, while similar sentences in Group 1F were significantly different only from ungrammatical sentences. Thus, the idea that every noun denoting a profession or a social role, even the most stereotypically male one, can be used to refer to a woman is already present in the mental grammar of Russian speakers (otherwise feminine agreement in Group 1 M would not differ from ungrammatical examples). If the profession is perceived as a stereotypically female, feminine agreement is expected almost as much as masculine agreement, even though the subject noun looks like a typical masculine noun.

## 3 Experiment 2

In Experiment 1, target nouns were the first words in a sentence, and we analyzed how the information associated with them, both morphological and extralinguistic, was used when processing the gender of the predicate. In Experiment 2, we added adjectives to the picture. Firstly, we wanted to analyze how different target nouns would be read after feminine and masculine adjective forms and how the gender of the adjective would be used to predict the gender of the predicate — depending on the stereotypes associated with the subject noun. Secondly, in Experiment 1, all sentences with target nouns were grammatical, although some of them could have sounded a little unusual. Experiment 2 included some ungrammatical examples, and we explored how this influenced processing depending on the other factors.

### 3.1 Participants

51 native speakers of Russian (20 male and 31 female) aged 18–45 took part in Experiment 2.

### 3.2 Materials

In this experiment, we analyzed only role nouns. We created 32 sets of target sentences in four experimental conditions, like (10a–d). The subject noun denoted a stereotypically female profession according to Garnham and Yakovlev’s (2015) study in one half of the sentences, and a stereotypically male profession in the other half. All sentences were seven words long and had the same syntactic structure: an adjective (feminine/masculine) – a subject noun – an adverb – a verb (in the past tense, feminine/masculine) – three words modifying the predicate. Like in Experiment 1, all subjects and predicates were in the singular, and all subject nouns did not have established feminine pairs. We decided to use verb forms rather than copulas and adjectives/participles to make the task easier and inserted adverbs between the subject and the predicate to give readers more time to process the combination of the adjective and the noun.

1. a. MM: *Sedoj pedagog družeski poxlopal vypusknika po pleču.*

grey-haired_M_ teacher friendly tapped_M_ graduate on shoulder
b. MF: *Sedoj pedagog družeski poxlopala vypusknika po pleču.*
grey-haired_M_ teacher friendly tapped_F_ graduate on shoulder
c. FF: *Sedaja pedagog družeski poxlopala vypusknika po pleču.*
grey-haired_F_ teacher friendly tapped_F_ graduate on shoulder
d. *FM: *Sedaja pedagog družeski poxlopal vypusknika po pleču.
grey-haired_F_ teacher friendly tapped_M_ graduate on shoulder
‘The grey-haired teacher friendly tapped the graduate on the shoulder.’

As we mentioned in the introduction, not only the MM and FF conditions, but also the combination of a masculine adjective and a feminine verb form (MF) is possible in Russian, and only the *FM combination is ungrammatical. Thus, three factors were manipulated: (i) the gender of the adjective; (ii) the gender of the predicate; (iii) gender stereotypes. We distributed target sentences across four experimental lists using the Latin square principle, adding 88 grammatically correct filler sentences that did not contain role nouns or common gender nouns. The full list of stimuli can be found at OSF.

### 3.3 Procedure

The procedure was the same as in Experiment 1.

### 3.4 Analysis

We analyzed participants’ question-answering accuracy and reading times. On average, participants answered only 6.5% of questions incorrectly (14.8% at most), so a breakdown of RTs into correct and incorrect question trials was not performed. Reading that exceeded a threshold of 2.5 standard deviations, by region and condition, were excluded (Ratcliff, 1993). In total, 2.7% of the data was excluded (at most 5.9% per region and condition).

Like in Experiment 1, we modeled the data with mixed-effects linear regressions in the *R* software (see text footnote 9) using the *lmer* function from the *lme4* package (Bates et al., 2015). To obtain *p* values from the *t* values given by the model, we used the *lmerTest* package (Kuznetsova et al., 2017). Random intercepts by participant and by item were included in the model. Tukey’s tests from the *multcomp* package (Hothorn et al., 2008) were used for *post hoc* comparisons. The condition (MM, FF, MF and *FM, with MM as the reference level) was treated as the fixed effect in different comparisons.

### 3.5 Results and discussion

**Figure 4:**
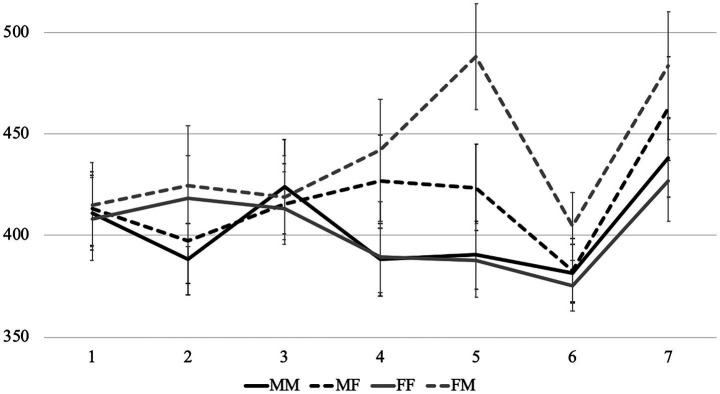
Experiment 2: Average word-by-word RTs (in ms) in the group F (with nouns denoting stereotypically female professions).

**Figure 5:**
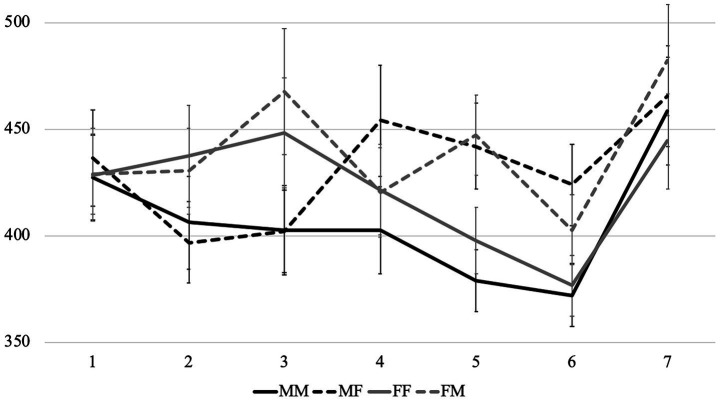
Experiment 2: Average word-by-word RTs (in ms) in the group M (with nouns denoting stereotypically male professions).

Average reading times per region in different conditions are presented in Figures 4, 5. First of all, we can see that the patterns in the sentences that contain subject nouns associated with stereotypically female and stereotypically male professions (we will further call them *Group F* and *Group M*) were very different. Therefore, below we analyze them separately, starting with the former.

We built two models with the condition as a fixed effect followed by pairwise comparisons between all conditions. We report the results of these comparisons below, while full outputs of all models can be found at OSF. Like in Experiment 1, there were no significant results in region 1, and we did not analyze the last region, so the results for regions 2–6 are discussed below. Since there were many significant effects in different regions, they are presented in Tables 2, 3.

**Table 2:** Experiment 2: pairwise comparisons between conditions for stereotypically female professions for regions 2–6.

Conditions	2	3	4	5	6
*FM vs. FF			*** β = 54.03, SE = 10.79, z = 5.01, *p* < 0.001	*** β = 102.26, SE = 10.61, z = 9.64, *p* < 0.001	*** β = 28.13, SE = 7.45, z = 3.78, *p* < 0.001
MF vs. FF			** β = 38.72, SE = 10.79, z = 3.59, *p* < 0.01	** β = 36.14, SE = 10.59, z = 3.41, *p* < 0.01	
MM vs. FF	No significant differences				
MF vs. *FM				*** β = −66.13, SE = 10.63, z = −6.22, *p* < 0.001	* β = −22.03, SE = 7.44, z = −2.96, *p* = 0.01
MM vs. *FM	** β = −36.79, SE = 11.29, z = −3.26, *p* < 0.01		*** β = −53.75, SE = 10.79, z = −4.98, *p* < 0.001	*** β = −98.70, SE = 10.62, z = −9.29, *p* < 0.001	** β = −24.08, SE = 7.42, z = −3.24, *p* < 0.01
MM vs. MF			** β = −38.43, SE = 10.79, z = −3.56, *p* < 0.01	** β = −32.57, SE = 10.60, z = −3.07, *p* < 0.01	

Significance levels are marked as follows: *p < 0.05, **p < 0.01 and ***p < 0.001.

**Table 3:** Experiment 2: pairwise comparisons between conditions for stereotypically male professions for regions 2–6.

Conditions	2	3	4	5	6
*FM vs. FF				*** β = 49.89, SE = 8.11, z = 6.15, *p* < 0.001	** β = 27.16, SE = 7.94, z = 3.42, *p* < 0.01
MF vs. FF	*** β = −41.11, SE = 10.77, z = −3.82, *p* < 0.001	*** β = −48.07, SE = 12.65, z = −3.80, *p* < 0.001	** β = 34.06, SE = 10.99, z = 3.10, *p* < 0.01	*** β = 44.90, SE = 8.06, z = 5.57, *p* < 0.001	*** β = 48.01, SE = 7.95, z = 6.04, *p* < 0.001
MM vs. FF	*** β = −31.64, SE = 10.77, z = −2.94, *p* = 0.01	*** β = −47.58, SE = 12.61, z = −3.77, *p* < 0.001			
MF vs. *FM	** β = −35.14, SE = 10.74, z = −3.27, *p* < 0.01	*** β = −64.42, SE = 12.63, z = −5.10, *p* < 0.001	* β = 30.07, SE = 11.11, z = 2.71, *p* = 0.03		* β = 20.85, SE = 7.94, z = 2.63, *p* = 0.02
MM vs. *FM		*** β = −63.92, SE = 12.60, z = −5.07, *p* < 0.001		*** β = −65.40, SE = 8.14, z = −8.03, *p* < 0.001	*** β = −29.66, SE = 7.98, z = −3.72, *p* < 0.001
MM vs. MF			*** β = −53.91, SE = 10.96, z = −4.92, *p* < 0.001	*** β = −60.42, SE = 8.09, z = −7.47, *p* < 0.001	*** β = −50.51, SE = 8.00, z = −6.32, *p* < 0.001

Significance levels are marked as follows: *p < 0.05, **p < 0.01 and ***p < 0.001.

In Group F, we can see that the readers slowed down after a feminine adjective compared to a masculine one, but most differences between conditions did not reach significance, only MM was significantly faster than *FM. Thus, although feminine agreement was grammatical, it still took slightly longer to process, like in Experiment 1. However, in the next region (the adverb) we did not observe not only any significant, but also any visible differences between conditions, i.e., this slow-down was very short-lived. Moreover, the readers successfully used the grammatical information on the adjective to process the gender of the verb: the MM and FF conditions had virtually identical reading times from region 4 (the verb) until the end of the sentence. In the MF and *FM conditions the readers reacted to the mismatch between the gender of the adjective and the verb, but, especially starting from region 5, the delay was especially pronounced in the ungrammatical *FM condition. As a result, the two matching conditions (MM and FF) differed significantly from the two mismatching conditions (MF and *FM) in regions 4 and 5, but at the same time, in regions 5 and 6, the *FM condition was significantly slower than the three others.

In Group M, the delay triggered by the feminine adjective in region 2 (the noun) was much larger than in Group F. Moreover, in Group F it disappeared in region 3 (the adverb), while in Group M, it only grew. Accordingly, all pairwise comparisons between conditions with feminine and masculine adjectives were significant in both regions, expect for MM vs. *FM in region 2. While in Group F, the readers immediately used the gender of the adjective to form expectations about the gender of the verb, in Group M, they could not do so — presumably, because the subject noun was strongly associated with male referents. It was so difficult for the readers to accommodate the mismatch between these stereotypes and the gender or the adjective that the cognitive load disrupted efficient processing of the upcoming words. As a result, in region 4 (the verb), the FF condition took virtually the same time to read as the ungrammatical *FM condition — they were both slower than MM, but were not significantly different from it. In the MF condition, the reading times were significantly longer than in the other three, and this picture persisted until region 6, although MF was grammatical, unlike *FM (only in region 5, the MF vs. *FM comparison did not reach significance). The *FM condition was also significantly different from MM and FF is regions 5 and 6, while the latter two did not differ significantly and eventually converged in region 6. Thus, the mismatch between stereotypicality and morphosyntactic information is resolved towards the end of the sentence, and a feminine adjective clearly helps to process a feminine verb form. Examples in which a feminine verb form follows a masculine adjective were especially difficult to process, although they are grammatical in Russian.

To conclude, there was a general preference for masculine agreement on the adjective in both groups, but it was much more pronounced in Group M. This result was parallel to Experiment 1 — in other words, the picture was similar both when the agreement controller followed the target and when it preceded it. After the grammatical information from the adjective was processed, the readers used it when processing the gender of the verb in Group F: they clearly expected it to be of the same gender as the adjective, although the MF combination is also grammatical in Russian. Ungrammatical *FM sentences took significantly longer to read than the others, as expected. In Group M, the influence of stereotypes was so strong that it disrupted these processes: the readers did not expect the feminine gender on the verb, even after a feminine adjective. As a result, the MF condition took longer to read than the others, including the ungrammatical *FM, while reading times for the FF condition converged with the MM only towards the end of the sentence.

## 4 Experiment 3

In Experiment 3, we collected acceptability judgements for target sentences from Experiment 2 to complement online data with offline data. We hypothesized that ungrammatical sentences would receive the lowest ratings, but grammatical sentences would also differ from each other, reflecting stereotypicality effects and the general preference for masculine agreement.

### 4.1 Participants

98 native speakers of Russian (33 male and 65 female) aged 18–52 took part in Experiment 3.

### 4.2 Materials

Materials were 24 sets of target sentences from Experiment 2 in four experimental conditions, like (10a–d) above. We also took 24 fillers from Experiment 2 to make materials more diverse. The number of stimuli and fillers was smaller than in Experiment 2 to make the task manageable. Target sentences were distributed across four experimental lists using the Latin square principle, while fillers were the same in all lists. The full list of stimuli can be found at OSF.

### 4.3 Procedure

The study was conducted via Google Forms. Participants were asked to judge the acceptability of the sentences on the 1 to 5 scale. The sentence order was randomized for each participant. We added two practice items before the main part of the experiment.

### 4.4 Analysis

We analyzed participants’ ratings with mixed-effects ordinal regressions in the *R* software (see text footnote 9) using the *clmm* function from the *ordinal* package (Christensen, 2023). Random intercepts and random slopes by participant and by item were included in the model. The first model reported below converged, while the second one did not, so we used only random intercepts for it. The *emmeans* package (Lenth, 2024) was used for *post hoc* comparisons.

The condition (MM, FF, MF and *FM) and stereotypicality were treated as fixed effects in different comparisons. We also included two-way interactions between the factors. For stereotypicality, we used contrast coding: 0 for ‘male’ roles and professions, 1 for ‘female’. When all four conditions were compared, MM was used as the reference level; when the MM condition was not included, the reference level was FF.

### 4.5 Results and discussion

Average ratings are presented in Table 4. We can see that sentences with masculine agreement both on the adjective and on the verb (MM) received the highest ratings — even when subject nouns denoted stereotypically female roles and professions. Ungrammatical *FM sentences had the lowest ratings, and this was not visibly affected by stereotypicality, unlike in Experiment 2. Sentences with the feminine agreement both on the adjective and on the verb (FF) or on the verb only (MF) were in the middle. Their ratings almost did not differ, with the latter being slightly better than the former, which does not correspond to online data — while reading, seeing a feminine adjective helped to form expectations about feminine agreement on the verb, so the FF condition was processed faster. Evidently, this faciliatory effect did not carry over to offline judgments. In these two groups, the effects of stereotypicality were more pronounced than in the other two groups.

**Table 4:** Experiment 3: average ratings across conditions.

Condition	Stereotypicality	Average rating	SD
*FM	F	1.5	1.0
	M	1.3	0.8
FF	F	3.1	1.5
	M	2.5	1.5
MF	F	3.2	1.6
	M	2.8	1.6
MM	F	4.6	0.8
	M	4.7	0.7

In the statistical analysis, we first built a model with the condition as a fixed effect — unlike in Experiment 2, we analyzed the whole dataset together because sentences with different stereotypes showed comparable patterns. We did not include the stereotypicality factor in this model because its role in different conditions was fundamentally different: stereotypically female roles and professions could be expected to improve ratings in the FF, MF and *FM conditions, but not in the MM condition, in which stereotypically male roles and professions should have been fine. In fact, they could have been expected to be rated much better, but average ratings in Table 4 indicate that this was not the case, demonstrating a strong general preference for masculine agreement. Therefore, we analyzed the former three conditions separately in the second model, taking stereotypicality into account.

The first model including the whole dataset with the condition as a fixed effect was followed by pairwise comparisons between all conditions, and we report their results below. Full outputs of all models can be found at OSF. Confirming the results of the descriptive analysis above, all conditions were significantly different from each other, except for the FF and MF (MM vs. FF: *β =* 4.92, *SE =* 0.54, *z =* 9.17, *p <* 0.001; MM vs. MF: *β =* 4.53, *SE =* 0.54, *z =* 8.47, *p <* 0.001; MM vs. *FM: *β =* 8.39, *SE =* 0.53, *z =* 15.94, *p <* 0.001; FF vs. *FM: *β =* 3.47, *SE =* 0.42, *z =* 8.27, *p <* 0.001; MF vs. *FM: *β =* 3.86, *SE =* 0.43, *z =* 8.88, *p <* 0.001). Thus, the ungrammatical *FM condition was the worst, but otherwise we could see a clear preference for masculine agreement — despite the fact that the FF and MF conditions are grammatical.

The second model was built to test the role of stereotypicality in the acceptability of feminine agreement in different conditions. Accordingly, it included only the conditions with feminine agreement; the condition and stereotypicality were treated as fixed effects. Subject nouns associated with male roles and professions significantly lowered the ratings (*β =* −0.87, *SE =* 0.30, *z =* −2.88, *p <* 0.01). The *FM condition differed significantly from the FF condition taken as the reference level, which we already know from the previous model (*β =* −2.85, *SE =* 0.18, *z = −*15.41, *p <* 0.001). Interestingly, the difference between MF and FF also reached significance (*β =* 0.39, *SE =* 0.15, *z =* 2.58, *p =* 0.01). Pairwise comparisons between conditions in the first model may be more reliable, but this result is still very telling and stresses the difference between online processing and offline acceptability judgements. Finally, the interaction between stereotypicality and the *FM condition was significant (*β =* 0.68, *SE =* 0.33, *z =* 2.08, *p =* 0.04), showing that in this condition, the effects of stereotypicality were overridden by ungrammaticality. The interaction between stereotypicality and the MF condition was not significant, as expected.

These results are interesting both from the extralinguistic and from the grammatical perspective. Although Russian allows using feminine agreement with nouns denoting roles and professions, native speakers are clearly hesitant to fully accept such sentences, especially when hindered by gender stereotypes. There is a clear general preference for masculine agreement — even for the roles and professions that Russian speakers are known to associate with women. In terms of the grammar, this is a curious example of grammatical sentences that are rated significantly lower than other grammatical sentences not for semantic or processing reasons, which are usually invoked in such cases, but because of some grammar-internal considerations, namely, because their subjects resemble masculine nouns.

## 5 Conclusion

In this paper we reported three experiments focusing on Russian nouns that denote professions and social roles. Historically, most nouns in this group were masculine, but in modern Russian they can refer to a woman (unlike any other animate masculine nouns), even when they have an established feminine pair, like *učitel’* ‘teacher_M_’ – *učitel’nica* ‘teacher_F_’. In the nominative, both masculine (‘formal’) and feminine (‘semantic’) agreement can be used in this case, while in oblique cases, only masculine agreement is grammatical. As we showed in the introduction, there are further grammatical restrictions on the possible agreement patterns. In particular, if we have attributive and predicative agreement, for example, an adjective and a verb, the former can be semantic only if the latter is also semantic.

This group is cross-linguistically unusual, so many authors discussed it in different theoretical frameworks (e.g., Asarina, 2009; Caha, 2019; Corbett, 1982, 1991, 2006, 2023; Lyutikova, 2015; Matushansky, 2013; Pesetsky, 2013; Privizentseva, 2024; Steriopolo, 2019; Steriopolo and Wiltschko, 2010). Acquisition of gender agreement in this group also attracted attention (e.g., Dizer, 2007; Dobrova, 2013; Rodina and Westergaard, 2012; Rodina, 2014; Tseitlin, 2000), while processing studies are still very few. Magomedova and Slioussar (2021) compared masculine and feminine agreement with different case forms in a word-by-word self-paced-reading study. The latter always took longer to process, even with the nominative case. Garnham and Yakovlev (2015) studied processing of predicative agreement and personal pronouns with role nouns, depending on gender stereotypes associated with them, but measured only sentence-by-sentence reading times and yes/no acceptability judgments of two-sentence passages.

The goal of the present study was to analyze online processing and offline acceptability of masculine and feminine agreement with these nouns, taking stereotypicality into account, like Garnham and Yakovlev (2015), but using more fine-grained measures: word-by-word reading times and acceptability on the 1 to 5 scale. We looked at predicative and attributive agreement. We believe that, firstly, our experimental results complement the picture emerging from theoretical studies. In particular, these studies describe different patterns as grammatical or ungrammatical, possible or impossible, while our experiments add a lot of nuances, which we summarize below. Secondly, these experiments are interesting in the light of the studies dedicated the effects of gender stereotypes in different languages.

As we showed in section 1.2, existing experimental studies focus on the choice between masculine and feminine role nouns and primarily on personal pronouns or noun phrases used anaphorically after role nouns. These studies demonstrated that a mismatch between gender stereotypes and the grammatical gender causes localized reading time delays and lowers acceptability. These effects may be more pronounced for masculine role nouns than for feminine ones. It was also noted that masculine role nouns used as generic plurals have a male bias. However, very little was known about the role of stereotypes in the processing of agreement. As it seems to us, the case of Russian role nouns is particularly interesting because in all other cases discussed in the literature, the choice of the grammatical gender depends on the presumed gender of the referent(s), while in Russian, both feminine and masculine agreement is possible for female referents of role nouns. However, as our experiments show, being possible does not mean being equally easy to process or being perceived as equally acceptable.

Let us discuss acceptability data from Experiment 3 first. We can see that sentences in the ungrammatical *FM condition (in which the attributive adjective is feminine and the verb is masculine) received the lowest ratings, while sentences in which all forms are masculine (MM) were rated the highest — independently of gender stereotypes associated with subject nouns. The FF and MF conditions were in the middle, and their ratings were significantly influenced by gender stereotypes. Thus, although feminine agreement is grammatical with role nouns, Russian speakers clearly do not accept it as readily as masculine agreement. Notably, MF examples were rated slightly higher than FF examples (this difference reached significance in one of the models), i.e., the less feminine agreement the better.

The preference for masculine agreement may be due to the very strong connection between genders and declensions that exists in Russian (the nouns in question belong to the declension IIa, which otherwise includes only masculine nouns). As we discussed in section 1.1, this is probably the reason for the absence of feminine agreement in oblique cases. Now we can conclude that this may also lower the acceptability of feminine agreement in grammatical sentences with nominative case. The downgrading of the ratings is especially pronounced in the sentences with stereotypically male roles and professions, in which feminine agreement is less expected. While the latter effect is probably semantic in its nature, otherwise this is a curious case when grammar-internal considerations (the connection between genders and declensions), rather than semantic or processing problems lower the ratings of fully grammatical sentences.

In many cases reported in the literature, offline acceptability data are parallel to online reading time data: the conditions that take longer to read are also rated lower (e.g., Fanselow, 2021). Data from Experiments 2 and 3 offer an interesting counterexample. The MF condition was rated slightly higher than FF. However, during online processing the readers were much faster with the sentences in which the gender of the adjective matched the gender of the verb (MM and FF). We do not have a definitive explanation for this phenomenon, but suppose that it is due to some general processing mechanisms. With the absolute majority of subject nouns, gender and number features should be the same on all agreeing elements, so we suppose that the parser may have adapted to this generalization. To shed more light on this question, it would be great to look at sentences with different word orders (for example, in Russian the verb may precede the subject).

In the sentences with stereotypically female professions, MM and FF conditions virtually did not differ once the information from the adjective was incorporated, while the *FM examples were the slowest. In the sentences with stereotypically male professions, feminine agreement on the verb was so unexpected after a masculine adjective that MF sentences even took longer to process than the ungrammatical *FM condition. Stereotypes also hindered processing of the FF examples, which converged with MM ones much later than in the sentences with stereotypically female professions.

In Experiment 1, there was only predicative agreement, so by looking at it and at the ‘adjective – noun’ combinations in Experiment 2, we can see how the readers react when they first see a target noun with a feminine or masculine agreement (when the controller follows the agreement target and when it precedes it). Masculine agreement was clearly preferred, but the effects were numerically small in the sentences with stereotypically female roles and professions, and did not reach significance in some comparisons. When sentences with different stereotypes were directly compared to each other, a significant interaction of the stereotypicality and grammatical gender was observed. Finally, let us note that stereotypicality effects were fully incremental, like in the previous studies (e.g., Banaji and Hardin, 1996; Garnham et al., 2002; Oakhill et al., 2005; Reynolds et al., 2006; Pyykkönen et al., 2010). This confirms that inferences based on stereotypes are made immediately and affect processing.

In Experiment 1, we could also compare target sentences to other sentences containing masculine or feminine animate subjects. Accordingly, only masculine or feminine agreement was grammatical with such subjects. All sentences with target nouns were processed significantly faster than ungrammatical sentences. However, only sentences with stereotypically male roles and professions significantly differed from other grammatical sentences with feminine agreement. We believe that these processing results, as well as acceptability results discussed above, nicely complement the portrait of role nouns drawn in various theoretical studies.

As for the directions for further research, apart from those outlined above, we would like to look at the processing of paired nouns. Using a (historically) masculine role noun, with feminine agreement or not, to refer to a woman is possible even when there is a feminine pair. But we do not know whether pairedness may influence reading times or acceptability judgements. Another interesting domain to explore concerns individual differences between participants: do their gender, age, or educational background influence the results? The phenomenon we study is not very novel in Russian (e.g., Muchnik, 1971; Graudina et al., 1976). Speakers of different genders and generations, with different education are familiar with it. Moreover, using feminine agreement with the role nouns of interest is not perceived as ‘feminist’ — an ideologically loaded debate revolves around the use of grammatically feminine role nouns. Therefore, we cannot say that any obvious differences may be expected, but some subtler patterns may emerge.^10^

## Data Availability

The datasets presented in this study can be found in online repositories. The names of the repository/repositories and accession number(s) can be found at: https://osf.io/wtu37.

## Footnotes

^1^There are several ways to transliterate Russian words from Cyrillic to Latin alphabet. In this paper, we use the so-called scholarly transliteration system.

^2^In case of other nouns denoting people, only a feminine noun can be used to refer to a woman. The majority of such nouns come in pairs. Several nouns are unpaired, like *mudrec* “sage_M_”, so one would have to use expressions like *mudraja ženščina* “wise woman” or *ženščina-mudrec* “woman-sage”.

^3^Garnham and Yakovlev also had stimulus passages with role nouns in plural in the first sentence and expressions *neskol’ko mužčin/ženščin* “some men/women” in the second sentence. However, they did not observe any clear effects in reading times, only lower ratings for passages referring to women, especially when role nouns were associated with male streotypes.

^4^Pronouns are also very interesting to study, but they received more attention in this field, and their choice depends exclusively on the extralinguistic factors, namely, on the perceived gender of the referent (which can be guessed both based on stereotypes and on gender agreement with antecedent nouns). Therefore, in the present study, we decided to focus on agreement processing.

^5^As we noted in the introduction, there may be some problems with this list, but we believe that may affect some details (how strong gender stereotypes associated with particular nouns are etc.), but not the big picture. Therefore, we selected role nouns with very clear preferences for male or female stereotypes.

^6^Acceptability of different examples is discussed only in Experiment 3, but we can note informally that sentences like (7b) sound unusual. As native Russian speakers, we would probably rather use an expression like *ženščina-mjasnik* ‘woman butcher’ in such cases, which unambiguously indicates the referent’s gender and requires feminine agreement.

^7^ https://osf.io/wtu37/?view_only=20bec09bab70483781fcdbd469206423

^8^ https://spellout.net/ibexfarm/

^9^ www.r-project.org

^10^We preliminarily analyzed data from our experiments adding age or gender as fixed effects (all our participants had similar educational background). More speakers with different demographic characteristics should be recruited to make any definitive conclusions. Firstly, we can say these characteristics do not affect the overall picture. As for subtler effects, we found that older people took longer to process ungrammatical sentences (in Group 2 in Experiment 1 and in the *FM condition in Experiment 2) and, more generally, sentences with grammatical mismatches (role nouns that resemble masculine nouns with feminine agreement in Experiment 1 and the MF condition in Experiment 2, which also received lower ratings from older people). Importantly, age never interacted with stereotypicality: mismatches associated with gender stereotypes were not costlier for older people, and no other differences emerged. If the generalization we observed can be confirmed, it would be important to find out whether this effect is specific for role nouns or more general. Notably, unlike the VF condition, the FF condition did not cause additional problems for older people. This may be an indication that the effect is not specific to role nouns with feminine agreement.
